# Retraction: Dye diffusion during laparoscopic tubal patency tests may suggest a lymphatic contribution to dissemination in endometriosis: A prospective, observational study

**DOI:** 10.1371/journal.pone.0339071

**Published:** 2025-12-18

**Authors:** The PLOS One Editors

The *PLOS One* Editors retract this article [1] because it was identified as one of a series of submissions for which we have concerns about compromised peer review and compliance with PLOS policy on Manipulation of the Publication Process.

MS and PG did not agree with the retraction. MN responded but expressed neither agreement nor disagreement with the editorial decision. AP, AS, GB, and GZ either did not respond directly or could not be reached.
